# Causes and Severity of Dentophobia in Polish Adults—A Questionnaire Study

**DOI:** 10.3390/healthcare9070819

**Published:** 2021-06-28

**Authors:** Dominika Furgała, Kinga Markowicz, Aleksandra Koczor-Rozmus, Anna Zawilska

**Affiliations:** 1 Student’s Scientic Society of the Department of Conservative Dentistry with Endodontics, Faculty of Medical Sciences, Medical University of Silesia, Pl. Akademicki 17, 41-902 Bytom, Poland; d.furgala20@gmail.com (D.F.); kingamarkowicz98@gmail.com (K.M.); 2 Department of Conservative Dentistry with Endodontics, Faculty of Medical Science, Medical University of Silesia, Pl. Akademicki 17, 41-902 Bytom, Poland; azawilska@sum.edu.pl

**Keywords:** dentophobia, dental anxiety, dental fear

## Abstract

Introduction: A phobia is defined as an irrational fear, the intensity of which is inadequate to the circumstances, and it leads to the avoidance of situations that trigger it. A person with dentophobia avoids dental treatment, even when the pain in the teeth or oral cavity exceeds their fear. Fear and anxiety are the most common emotional states experienced by patients in dental offices. The aim of the study was to determine the following: the probable causes of dentophobia, which procedures patients fear the most, and the subjective feeling of fear on a point scale. Materials and Methods: The research was conducted in the form of an anonymous online questionnaire and its printed equivalent. The survey was available from 23 January to 16 March 2020, and it was addressed to adult respondents. In total, 130 respondents and 102 dental students took part in the survey. Results: It is worthwhile emphasizing that the main reason for dentist visits (for almost one-third of men and almost one-fifth of women surveyed) is a toothache. Periodontal problems (2.2%), tooth extractions (1.8%), and prosthetic consultations (0.9%) were relatively rare reasons for the respondents to visit a dentist. The vast majority of respondents see the cause of dentophobia as being related to their previous bad experiences. The second most frequently chosen answer is anxiety, which is caused by the sounds of dental apparatus. Conclusions: Pain is the most common reason given for fear of visiting a dentist, as well as the cause of dentophobia. Pain reduction can positively affect the frequency of visits to a dentist, thus, improving the condition of the oral cavity of patients.

## 1. Introduction

Dentophobia is described as an intense fear of dental procedures performed by a doctor, or of doctors themselves, and it lasts until the treatment is discontinued [1]. It is a type of paralyzing fear that makes it difficult for patients to make decisions regarding a possible visit to a dentist’s office or whether to cancel the visit. The causes of this type of fear are rooted in negative childhood experiences or in the fear of pain [2]. Anxiety leads to deteriorating oral health (including professional hygiene procedures) and implies that patients go to a dentist only when they begin to feel severe pain, which is related to postponing appointments and not treating carious lesions voluntarily [1].

Fear and anxiety are the most common emotional states experienced by patients in dental practices. Although the definitions of the two terms differ from each other, they are often used as synonyms [3]. Anxiety is a mental state associated with a sense of threat that cannot be precisely defined [1]. Dental anxiety occurs in patients and also in healthcare professionals. It is an individual reaction of varying severity and a response to a situation in the office that may cause stress. Its terms are defined in various ways, such as anxiety, fear, and phobia, the definitions of which vary [4]. Fear is a natural reaction of the body to danger, but it can be a strong and unpredictable response. In a dentist’s office, fear can be triggered by various stimuli, such as an anesthetic needle or the sound of a turbine [5]. Phobia is a stronger feeling. It is defined as irrational fear, the intensity of which is inadequate to the circumstances, and which can lead to an avoidance of the situations that trigger it. A person suffering from dentophobia avoids (or even runs away from) dental treatment until the pain in their teeth or oral cavity exceeds the fear itself [6].

Fear makes the treatment a very uncomfortable experience. It also disturbs the doctor–patient relationship, accelerates the patients’ breathing and heart rate, and can cause post-treatment complications; in effect, it causes the patient to avoid treatment at all costs. According to many authors, dental anxiety depends on several factors, such as gender, age, education, or the condition of the oral cavity. Such a negative attitude by a patient to treatment at the beginning of a dental procedure increases anxiety and fear and, consequently, may lead to complications both during and after treatment [2].

The etiology of fear of a dentist works on many levels, and specialists dealing with it are still trying to understanded it in greater depth. Rachmann presented three possible mechanisms for acquiring fear, which were: (1) based on exposure to information that causes fear, (2) related to the observation of other people’s behavior, and (3) based on experiences related to painful and unpleasant experiences during a visit to a dentist [7]. A Fiesta survey of Seattle residents in 1986 showed that fear of a dentist was one of the most frequently indicated responses by participants, right behind fear of heights and accidents [8].

According to the guidelines by the American Academy of Pediatric Dentistry (AAPD), a dentist should take into account the assessment of a child’s dental fear and the level of their cooperation when planning treatment. The ideal assessment method according to AAPD should be accurate, should take into account the cognitive and linguistic limitations of a child, and should be easy to perform in practice [9].

Both subjective and objective methods are used to assess the fear associated with dental treatment. Despite the fact that there are various tools for the subjective assessment of anxiety levels using behavioral, projective, or psychometric techniques, there is still no method that strikes the right balance. Among the subjective methods are self-assessment questionnaires, which themselves have significant disadvantages. Objective methods are based on the physiology of the organism [9].

### Purpose of the Study

The aims of this study were to determine the following: (1) the probable causes of dentophobia, (2) which procedures patients fear the most, and (3) the subjective feelings of fear on a point scale. Moreover, an attempt was made to present methods for reducing the stress of patients, as well as methods to categorize how the doctor–patient relationship influences the success of the visit and what procedures patients follow when choosing a dentist. The participation of dental students helped to confirm or deny the hypothesis that students feel less fear in relation to visiting a dentist; this was considered in conjunction with their year of study.

## 2. Materials and Methods

This study was conducted in the form of an anonymous online questionnaire and its printed equivalent. The survey was available from 23 January to 16 March 2020, and it was addressed to adult respondents. In total, 130 participants took part in the survey (50.8% (66) were women, 49.2% (63) were men). The group of respondents came from different regions of Poland and represented several age groups (see Table 1). A second group of participants included 102 dental students in the Faculty of Medical Sciences at the Medical University of Silesia (see Table 2).

The questionnaire consisted of 18 single-choice questions. The form included standard sociometric questions about gender and age. The next part of the survey included questions about the last visit to a dentist’s office, the frequency of such visits, and the reasons for visiting the doctor. The following questions were mainly related to the topic of dentophobia. The respondents answered questions about their knowledge of the concept of dentophobia, what might cause it, and their own experiences and feelings, for example, what causes them to fear visiting a dentist or whether they have ever canceled a visit because of anxiety. The respondents also commented on what they consider when choosing a dentist, as well as the best way to overcome fear of an appointment. One of the objectives of the questionnaire was to determine feelings of anxiety on a scale from 0 to 5. The questions also concerned the occurrence of somatic symptoms related to the fear of visiting a dentist, as well as feeling fear in specific situations.

An additional section was added for dentistry students, which included questions about their studies (year of study) and the methods used to facilitate the course of their dental visits. The results were collected and analyzed in the form of figures and tables.

The data were collected anonymously, and the procedure was approved by the Ethical Commission of the Medical University of Silesia on 16 October 2018 (reference number KNW/0022/KB1/79/18). All the participants indicated their consent to participate in the survey when completing the questionnaire.

### Statistical Procedures

Statistical analyses were performed using Statistica Version 9.0 (StatSoft, Tulsa, OK, USA). For the assessment of the statistical significance of differences between participants and dental students in terms of gender, we used a non-parametric independence χ^2^ test. In the case of small samples, statistical analysis was supplemented with a Fisher’s exact test. The Mann–Whitney test was used to assess the differences in values from the dental anxiety scales in terms of respondents’ gender. The significance level *p* was set to 0.05.

## 3. Results

### 3.1. Respondents

The non-parametric χ^2^ independence test did not show any statistically significant differences in terms of respondents’ gender (χ^2^ = 4.06, *p* = 0.29).

Table 3 shows how long ago the patients visited a dentist, how often they usually visit the office, and what the most common reason was for them visiting a dentist.

As the table shows, the majority of both genders had their last visit up to 6 months ago. There were no statistically significant differences observed between the genders (χ^2^ = 4.03 *p* = 0.25). In total, 43.1% of respondents visit a dentist once every 6 months; more men than women declare this to be their frequency of visits. However, the difference was not statistically significant (χ^2^ = 3.59, *p* = 0.46). Carrying out control visits at six-month intervals is one of the therapeutic recommendations for patients with a low and moderate risk of caries, according to CAMBRA (Caries Management by Risk Assessment) [3]. As for the reasons for visits to a dental office, results vary by gender; however, for 55.7% of men and for 43.5% of women, an oral cavity check represents the most cited reason. It is worthwhile paying attention to the cases when the reason for a visit is a toothache; here, almost one-third of the surveyed men considered it the main reason for visiting a dentist as compared with less than one-fifth of the surveyed women. Periodontal problems (2.2%), tooth extractions (1.8%), and prosthetic consultations (0.9%) were relatively rare reasons for the respondents to visit a dentist’s office. The reason for such a result is probably the fact that a large number of respondents are young people who are not yet affected by these conditions. The reported differences between men and women were not statistically significant (χ^2^ = 4.20, *p* = 0.24). The vast majority of the respondents were familiar with the concept of dentophobia, and no significant differences were found between men and women (χ^2^ = 2.57, *p* = 0.10).

Table 4 shows how the respondents answered the question about the causes of dentophobia. The vast majority of respondents see the cause of dentophobia as being related to previous bad experiences. The second most frequently chosen answer is anxiety, which can be caused by the sounds of dental equipment. Significant differences were not observed (χ^2^ = 4.20, *p* = 0.24). It is worth mentioning that factors such as high-frequency noise or the vibration of a drill can cause fear in children [5]. When referring to a dentist’s attitude, it should be remembered that the personality traits that facilitate a dentist’s work include a willingness to help, being interested in people’s well-being, a tendency to reflect on one’s actions, patience, and the general culture of the practice [5].

The next question concerned the factors that most often cause anxiety in patients. The results are presented in Table 5. The χ^2^ test did not show any significant differences between the genders (χ^2^ = 8.47, *p* = 0.076).

The respondents were asked about the usefulness of explaining the details of the procedure and how to build the doctor–patient relationship (see Table 6). In both cases, the vast majority admit that a friendly conversation may have a positive impact on the course of the visit and greater treatment success. However, a statistically significant difference between women and men was found (χ^2^ = 4.21, *p* = 0.04). Statistical analysis was completed by a Fisher’s exact test (*p* = 0.01). Building a good doctor–patient relationship was not as important for men as it was for women. Informing a patient about the treatment and how it will proceed is important in reducing said patient’s level of fear. It is important to choose the right vocabulary when dealing with a patient [5].

Dental anxiety can cause frequent changes of appointments, cancellations, or the avoidance of dentist visits. Such behavior by a patient is sometimes conditioned by the motivational conflict between striving and avoiding. It occurs when the action taken has both positive and negative meanings for the patient. Pain or disease induces a patient to visit a dentist, but the fear is often so strong that it causes reluctance to contact the doctor [5]. In total, 21 respondents (9.3%) admitted that they had canceled a dentist appointment; the rest did not experience such an incident. In addition, when asked about feeling anxious, 40.4% of the respondents replied that they did not feel any discomfort during visits, 26.2% felt fear before and during the visit, 20.8% felt fear during the visit, and 12% were anxious before the visit.

More than half of the respondents (51.1%) believe that fear of visiting a dentist can be overcome by finding the right dentist who shows empathy and can explain each stage of the procedure. A total of 24.9% of respondents believe that the elimination of anxiety is possible in a well-adapted and modern office that provides access to atraumatic treatments. It is worthwhile mentioning that, in the waiting room, there should be brochures and other materials that relate to the activities and methods used by a dentist during a dental treatment. Their content should reduce fear and anxiety in patients and mobilize treatment [5]. A total of 12.9% of respondents believe that anxiety decreases when anesthesia is used at each visit, while 7.1% say that it is necessary to inform their doctor about the problem of dentophobia. Only 4% of the respondents said that visiting a loved one helps them to overcome anxiety.

It is also worthwhile paying attention to what procedures patients follow when choosing a dentist. In total, 75% of respondents followed the opinion of family and friends, while 10.2% of the respondents used internet forums (e.g., well-known doctors). The reputation of a dental clinic is important for 8.9% of our respondents, and the price of the service is important for 4.4%.

### 3.2. Dental Students

The non-parametric χ^2^ independence test did not show any statistically significant differences in terms of the dental students’ gender (χ^2^ = 0.15, *p* = 0.63).

As shown in Table 3, most of both genders had their last visit up to 6 months ago. There were statistically significant differences observed between the genders (χ^2^ = 6.46 *p* = 0.03). Unfortunately, male dental students more often answered that they visited a dentist over a year ago; they indicated that they went for a check-up every two years (χ^2^ = 10.27, *p* = 0.01) (see Table 3 and Table 4).

Male dental students believe that fear of visiting a dentist can be overcome by finding the right dentist who shows empathy and can explain each stage of the procedure (χ^2^ = 2.65, *p* = 0.03). In contrast to male dental students, female dental students both rely on the opinions of family and friends and use internet forums and other sources when choosing a dentist (χ^2^ = 4.61, *p* = 0.03).

No significant differences were found between dental students in terms of gender in relation to the factors that most often caused dental anxiety (χ^2^ = 0.85, *p* = 0.83) (see Table 5).

**Figure 1:**
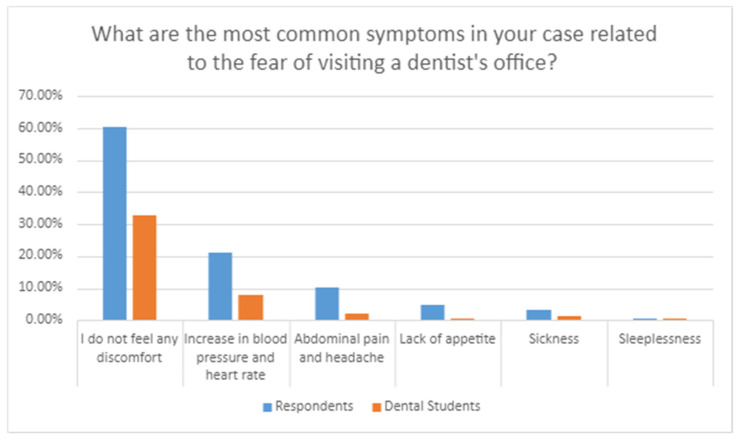
Types of symptoms appearing in a patient before visiting the office.

Figure 1 shows the most common symptoms in patients before visiting a dentist. In total, 10.2% of respondents reported that they feel abdominal pain and a headache before visiting a dentist. For 20.2% of participants, the fear of visiting a dentist manifests itself in increased blood pressure and a higher heart rate. However, 60.5% of respondents do not feel any discomfort associated with visiting a dentist.

### 3.3. Dental Ankiety Scale

Table 7 shows how patients assess their anxiety and stress before visiting a dentist. Less than half of the respondents did not feel any negative emotions in connection with visiting a dentist’s office.

Table 8 shows the level of fear experienced by dental students (the number in brackets is the percentage of all surveyed students). According to the survey, half of the surveyed students (54.9%) declared that they are not afraid of visiting a dentist.

**Figure 2:**
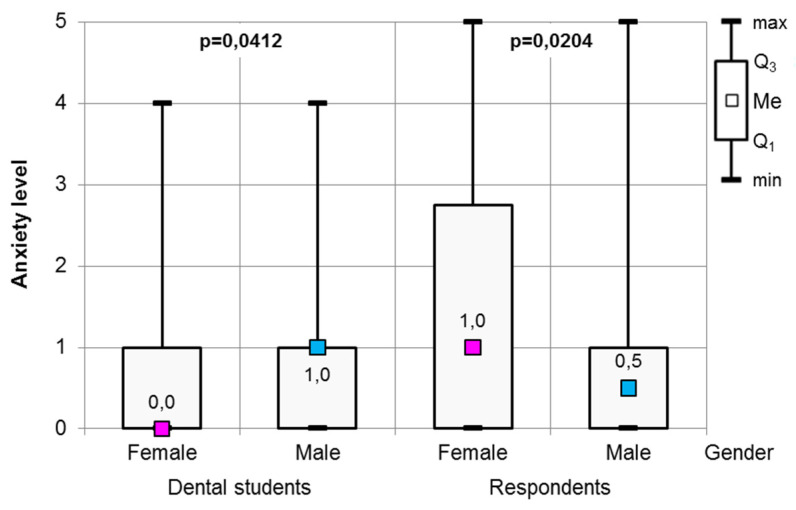
The results of the Mann–Whitney test for dental anxiety scale in terms of participants’ gender.

However, in the case of the dental anxiety scale, statistically significant differences were observed between the genders in both respondents and dental students. Female dental students were less afraid of visiting a dentist than male dental students (Mann–Whitney test, *p* = 0.0412). In contrast, female respondents felt higher levels of dental anxiety than their male counterparts (Mann–Whitney test, *p* = 0.0204) (see Figure 2).

## 4. Discussion

It is estimated that the fear of treatment in a dental office affects 6–15% of the world’s population, both adults and children. This fear is a great limitation to treatment, as ignoring control visits and avoiding systematic treatment, as well as appearing in a dental office only in cases of pain, are detrimental to overall oral health. Patients with this type of fear cause difficulties for their doctors, as they require more attention and cancel their visits. During the treatment of such individuals, doctors themselves experience additional stress [10].

The age range of the respondents in our first study was 18–55, and the range in the second study [11] was 18–80 (mean 44 years old). A similar age range was found in studies by Dobros et al. [12] (18–70, with a mean of 40) and Appukuttan et al. [13] (20–70, with a mean of 32). A slightly smaller age range was found in a study by Obeidat et al. [14], ranging from 18 to 65 years (with a mean age of 53 years).

A similar study on dentophobia was conducted by Czerżyńska et al. [1] in 2017, where the study group consisted of 330 patients at the Specialist Outpatient Clinics of the University Teaching Hospital in Białystok (Poland). In our survey of 232 people, 67.1% of respondents declared that they last visited a dentist up to 6 months ago. A slightly lower result (56.4%) was provided by the authors of the study mentioned above. When comparing the sexes, the number of women and men visiting a dentist’s office, being the target of the control tests over the last 6 (51.6% women and 60.6% men) and 12 months (19.4% women and 24.6% men), is similar. This is in contrast to the above studies, in which women visited a dentist for control more often (76.0 vs. 54.4%) [11]. The studies both showed similar results in the frequency of follow-up visits. In total, 46.0% of the surveyed women say that they visit a dentist once every 6 months as compared with 42.7% of women in the study by Czerżyńska et al. [1]. We observed a slightly different situation among the surveyed men, i.e., 32.8% vs. 19.4%. In the case of studies [1], a statistically significant correlation was found between sex and the frequency of visits to a dentist (*p* < 0.001). Women more often visited a dentist regularly every 6 months [1]. We also observed a similarity with respect to the most frequently quoted reason for a visit, i.e., a control visit (53.3% of the responses in our study and 50.6% of the responses in the Czerżyńska study). The second most common reason for a visit, as declared by the respondents in both studies, is a toothache.

A study by Krufczyk [11] showed a similarity regarding the main cause of fear reported by 215 patients; according to 60% of respondents, pain and unpleasant experiences from the past were primary sources of fear. In the cited study, more women than men declared visiting once every 6 months, or at least once a year. In our analysis as well as in the study by Krufczyk, the respondents recognized that the personality traits of a dentist are important and can help reduce the stress caused by a visit [11]. From the studies on the cause of dentophobia, we obtained similar results to those in a study by K. Sopińska [6]. In our study, 68.4% of respondents indicated that their fear began in childhood due to negative experiences related to dental treatment; in [6], a similar cause of dentophobia was indicated by 85% of respondents [6].

In the cited study [1], the fear rating scale was also used (where grade 1 meant anxiety and nervousness and grade 5 meant intense fear that made it impossible to visit). The scale did not include grade 0, as used in our study. In Czerżyńska’s analysis, both women and men most often defined the degree of their anxiety as Grade 2 (nervousness) while, in our study, Grade 1 was most frequently chosen (anxiety, nervousness); these responses omitted the selection of Grade 0, meaning no fear. In a questionnaire in the study by M. Krufczyk, 7.1% of women chose the intensity of fear as Grade 1, and 26.8% of women rated their fear as Grade 2 (nervousness). Grade 5 was chosen by 21.5% of women. Among men, Grade 1 was declared by 6.5%, Grade 2 was declared by 36.9%, and Grade 5 was chosen by 13.0% of men [11].

In total, 51.1% of respondents considered the choice of an empathetic and professional dentist to be the key to overcoming the fear of an appointment. According to the author, patients in pain require immediate care, as well as an appropriate approach and selective vocabulary. For patients, it is essential to maintain a good doctor–patient relationship (94.7% of respondents). For the respondents in a study by E. Gruz [2], the most important thing was the dentist’s professionalism (44% of the respondents), as well as a pleasant and friendly atmosphere in the dentist’s office (42%). The significant role of empathy and a good dentist–patient relationship, which should be part of the service, was also confirmed by other studies [15,16,17,18]. According to our survey, the fear of treatment is felt by 63.6% of patients, while 36.4% say that they are not afraid of dental treatment. According to research [6], fear of dental treatment affects anywhere from 16% to 24% of adults in various populations, and 12% to 16% of them suffer from dentophobia [6].

Predicting pain and troublesome or intensive treatment are among the most frequent sources of fear [5]. Pain can be reduced by using various anesthetic techniques (using a surface anesthetic at the puncture site or depositing a drop of anesthetic through the puncture) [7]. Computer-controlled anesthesia devices usually look less threatening and more aesthetic, and therefore are better accepted by patients. In addition, computer control and limited injection rates reduce a patient’s discomfort. These systems include The Wand, Sleeper One, Quick Sleeper, or Anaject, among others [5].

In terms of overcoming anxiety and reducing stress, finding a good (reliable) dentist is a significant factor. According to our research, 51.5% of respondents indicated this answer. In the research conducted by Krufczyk, the same answer was indicated by about 60% of people. In all, 24.9% of people declared that visiting a modern dental office can help with reducing stress. Quick performance of the procedure and modern equipment were the second and the third most frequent answers [11].

A study by Leutgeb, Ubel, and Schienle [19] showed that, regardless of gender, people suffering from dentophobia show an accelerated heart rate when viewing photographs of dental treatment as compared with a control group that does not experience dental anxiety. This symptom is a clear reflection of the fear that patients feel. The results of our survey show that patients with dentophobia state an increase in blood pressure and heart rate as the most common symptoms of fear associated with visiting a dentist.

In our study, the sound of a drill was identified as the second most common cause of dentophobia, and the second aspect that respondents feared most during treatment. A study conducted by Boyle et al. [20] aimed at characterizing the group of patients referred for treatment under sedation. Among these patients, one of the five most frequently reported treatment concerns was the sound of dental drills.

The aims of studies by Humphris et al. and Jeddy et al. [21,22] were to determine whether an earlier traumatic experience may influence the perception of dental anxiety. The studies confirmed the thesis and, among the previous unpleasant experiences reported by patients, tooth preparation, helplessness during treatment, injections, a lack of understanding from the dentist, and information in the media about dental treatment were all significant sources of anxiety. Although our study does not address the question of what specific experiences may be the cause of dentophobia, the majority of respondents (68.4%) believe that it is caused by experiences associated with pain.

Humphris’s [21] study results were also confirmed by A. J. van Wijk et al. [23], showing that anxious patients with previous negative experiences may feel more intensive pain during anesthesia, tooth extraction, and other dental procedures. The study results showed that people with severe anxiety had a lower pain threshold as compared with patients without dental phobia, which corresponded with the results of our research regarding the correlation between anxiety and pain.

In addition, a dental questionnaire by Arjen J. Van Wijk and Johan Hoogstraten [24], which corresponded to the general FPQ-III for assessing pain fear, showed that patients who were asked to indicate the degree of anxiety or fear that they may experience during certain very painful events reported that they were most fearful of a toothache. The aforementioned conclusions show a similarity with our study.

## 5. Conclusions

Pain is the most commonly cited reason for fear of visiting a dentist, as well as a cause of dentophobia. Pain reduction can positively affect the frequency of visits to a dentist, thus, improving the condition of patients’ oral cavities.

The study among dental students shows that half of them do not feel anxious about visiting a dentist. To summarize, fear of a dentist is very individual, and education in the field of medicine and dentistry has little effect on the level of fear experienced.

More attention should be given in the curriculum to prepare students to deal with patients with dentophobia. An equally important aspect, in the case of patients with dental anxiety, is an appropriate level of empathy, which should in any case characterize dentists. This urgency should also be included in the curriculum.

## Figures and Tables

**Table 1:** Characteristics of the study group.

Age	Number of Respondents (% of Respondents)	Number of Women (% of Respondents)	Number of Men (% of Respondents)
18–24	83 (63.9%)	35 (26.9%)	48 (36.9%)
25–34	26 (20%)	17 (12.9%)	9 (6.9%)
35–44	14 (10.7%)	10 (7.7%)	4 (3.0%)
45–54	7 (5.4%)	4 (3.0%)	3 (2.3%)
>55	0	0	0
In total:	130	66 (50.8%)	64 (49.2%)

**Table 2:** Characteristics of a selected group of dental students.

Year of Studies in the Field of Dentistry	Women (% of All Students)	Man (% of All Students)	Overall
1st	11 (10.8%)	3 (2.9%)	14 (13.7%)
2nd	21 (20.6%)	8 (7.8%)	29 (28.4%)
3rd	25 (24.5%)	8 (7.8%)	33 (32.3%)
4th	13 (12.7%	5 (4.9%)	18 (17.6%)
5th	7 (6.9%)	1 (0.9%)	8 (7.8%)
In total:	77 (75.5%)	25 (24.5%)	102

**Table 3:** Dates, frequency, and reason for visits to a dentist’s office among genders.

**The Date of the Last Visit**	**% Women**	**% Men**	**% Female Dental Students**	**% Male Dental Students**	**% Total**
Up to 6 months ago	51.6%	60.6%	81.8%	76.0%	67.1%
6–12 months ago	19.3%	24.6%	15.6%	8.0%	18.2%
Over a year ago	22.6%	9.8%	2.6%	12.0%	11.1%
Over a two years ago	6.4%	4.9%	0	4.0%	3.6%
**The Frequency of Visits**					
Once every 6 months	19.4%	32.8%	67.5%	52.0%	43.1%
Once a year	41.9%	29.5%	27.3%	20.0%	31.1%
Once every two years	19.4%	19.7%	2.6%	16.0%	13.3%
Once every 3 years or more seldom	6.4%	4.9%	0	0	3.1%
Lack of visits	12.9%	13.1%	2.6%	12.0%	9.3%
**The Reason for the Visit**					
Check-up visit	43.5%	55.7%	62.3%	44.0%	53.3%
Hygienic treatment	6.4%	4.9%	24.8%	20.0%	13.8%
Toothache	32.3%	32.8%	7.8%	20.0%	22.8%
Periodontal problems	6.4%	1.6%	0	0	2.2%
Making dentures	3.2%	0	0	0	0.9%
Teeth extractions	3.2%	0	2.6%	0	1.8%
Other disturbing conditions	4.8%	4.9%	2.6%	16.0%	5.3%

**Table 4:** The most common cause of dentophobia.

The Date of the Last Visit	% Women	% Men	% Female Dental Students	% Male Dental Students	% Total
Bad past experience of pain sensation during treatment	66.1%	52.5%	77.9%	84.0%	68.4%
Anxiety caused by the sound of a drill or other apparatus	16.1%	18.0%	14.3%	4.0%	14.7%
Unpleasant smell of the dentist’s office	1.6%	3.3%	1.3%	4.0%	2.2%
Deprived of empathy, rude and unreliable doctor	1.6%	8.2%	5.2%	4.0%	4.9%
Shame caused by the condition of the teeth	9.7%	3.3%	1.3	0	4.0%
Do not know what dentophobia is	4.8%	14.7%	0	4.0%	5.8%

**Table 5:** Patient fears related to dental treatment.

The Triggering Factor of Anxiety	% Women	% Men	% Female Dental Students	% Male Dental Students	Total %
The pain itself	33.9%	26.2%	33.9%	26.2%	30.2%
The sound of dental equipment	8.0%	16.4%	8.1%	16.4%	8.9%
Crossing the threshold of a dentist’s office	17.7%	8.2%	17.7%	8.2%	7.1%
Root canal treatment	11.3%	3.3%	11.3%	3.3%	6.2%
The moment of administration of anesthesia or its absence	6.4%	1.6%	6.4%	1.6%	4.9%
Tooth extraction	3.2%	8.2%	3.2%	8.2%	4.9%
Tooth root resection	0	0	0	0	0
I am not afraid of treatment	19.3%	36.1%	19.3%	36.1%	36.4%

**Table 6:** Patient assessment regarding the explanation of the treatment and purpose of the procedure, as well as the doctor–patient relationship.

	Women	Men
In your opinion, is it helpful for the patient to overcome the fear of visiting a dentist by explaining what the procedure will involve and what it aims to do?	Yes 87.1% No 12.9%	Yes 98.4% No 1.6%
Does building a good doctor–patient relationship have a great impact on overcoming the fear of visiting a dentist’s office?	Yes 79.0% No 20.9%	Yes 83.6% No 16.4%

**Table 7:** Assessment of anxiety before visiting a dentist between the genders.

Scale	Women	Men	Overall
0—no feeling of stress or fear	17 (27.4%)	28 (45.9%)	45 (36.6%)
1—anxiety, nervousness	22 (35.5%)	19 (29.5%)	41 (33.3%)
2—nervousness	5 (8.0%)	3 (4.9%)	9 (7.3%)
3—nervousness (trembling hands, stress at the thought of visiting)	10 (16.1%)	3 (4.9%)	13 (10.6%)
4—fear with symptoms (e.g., stomach aches and headaches)	6 (9.7%)	5 (8.2%)	11 (8.9%)
5—long-term fear that makes it impossible to visit	2 (3.2%)	2 (3.3%)	4 (3.2%)

**Table 8:** Assessment of anxiety before visiting dentist among dental students.

Scale	Female Dental Students	Male Dental Students	Overall
0—no feeling of stress or fear	46 (45.1%)	10 (9.8%)	56 (54.9%)
1—anxiety, nervousness	24 (23.5%)	11 (10.8%)	35 (34.3%)
2—nervousness	3 (2.9%)	2 (1.9%)	5 (4.9%)
3—nervousness (trembling hands, stress at the thought of visiting)	3 (2.9%)	1 (1.0%)	4 (3.9%)
4—fear with symptoms (e.g., stomach aches and headaches)	1 (1.0%)	1 (1.0%)	2 (1.9%)
5—long-term fear that makes it impossible to visit	0	0	0

## Data Availability

The data presented in this study are available on a reasonable request from the corresponding author.

## Abbreviations

The following abbreviations are used in this manuscript:

AAPD	The American Academy of Pediatric Dentistry
CAMBRA	Caries Management by Risk Assessment
FPQ-III	Fear of Pain Questionnaire
